# Breast cancer survivorship in South Africa: A holistic primary care approach

**DOI:** 10.4102/safp.v68i1.6200

**Published:** 2026-01-07

**Authors:** Mareike Rabe, Nita M. Robinson

**Affiliations:** 1 Vita Oncology, Cape Town, South Africa

**Keywords:** breast cancer survivorship, primary care, hormone therapy, psychosocial support

## Abstract

As breast cancer incidence increases and survival improves, an increasing number of breast cancer survivors (BCS) require long-term follow-up and support. Primary care providers play a central role in maintaining continuity of care, promoting endocrine therapy adherence, managing complications such as lymphoedema and supporting psychosocial well-being. This article aims to provide a structured approach to breast cancer survivorship care, aligned with national cancer care policies and guidelines and international best practices, focused on primary care integration. A case vignette aims to bridge theory and practice, contextualise decision-making and encourage patient-centred care as it relates to breast cancer survivorship in the South African context.

## Introduction

South Africa’s (SA) breast cancer burden continues to rise, and although advancements in early detection and treatment have improved survival, the country still has a high breast cancer mortality rate, likely because of factors such as low screening rates, lower access to health care and a higher incidence of triple-negative breast cancer in patients of African ancestry.^1^ Breast cancer is the leading cancer among South African women across all population groups, excluding non-melanoma skin cancers.^2^ Breast cancer accounted for 23.2% of all cancers diagnosed in women between 2009 and 2019. Deaths because of breast cancer increased by 42.4%, from 2 665 in 2008 to 3 796 in 2018.^3^ Although breast cancer in men is rare, accounting for up to 3% of cases in SA, it remains a critical yet often overlooked aspect of survivorship care.^4^ Treatment approaches for male breast cancer largely mirror those used in women, but there is a paucity of data of men’s experiences, perceptions and needs related to their cancer journeys.^4,5^ There is a need for more inclusive, gender-sensitive support from health care providers who deal with male breast cancer survivors (BCS).^5^

Breast cancer survivorship refers to the phase of care that begins at the time of diagnosis and continues through the remainder of a person’s life, regardless of whether one is still living with cancer or in remission. It encompasses not only the physical recovery from cancer and its treatment, but also the emotional, psychological, social and financial impacts that persist long after active treatment may have ended.^6,7^ This holistic view is especially relevant in SA, where primary care providers (PCPs) are increasingly responsible for long-term follow-up, endocrine therapy support and psychosocial care.

Survivorship care spans more than disease surveillance; it includes managing late complications, improving quality of life and coordinating health maintenance. With specialist services stretched, PCPs are uniquely placed to provide accessible, long-term follow-up.

## Case vignette

Ms. R is 65 years old. She presents to her local clinic for a consultation and script renewal of her anti-hypertensive treatment. She reports having undergone left-sided breast cancer treatment for a hormone receptor positive (HR+) 3 cm lesion without lymph node involvement at a tertiary centre 8 years ago. Her treatment consisted of:

- Neo-adjuvant chemotherapy.
- Breast conserving surgery and sentinel lymph node biopsy.
- Radiation therapy.
- Aromatase inhibitor (AI): letrozole, which she is currently still taking.

She requests further breast cancer follow up at her local clinic as she finds attending appointments at the tertiary centre cumbersome. Given that she feels well, she questions the need for frequent visits to the tertiary care centre. In approaching her care, it is essential to adopt a comprehensive and patient-centred strategy, considering the pertinent clinical and psychosocial issues that may influence ongoing management.

## The breast cancer survivorship journey: Initial care

Many BCS have undergone multimodal treatments provided by multidisciplinary teams (surgeons, medical and radiation oncologists, radiologists, pathologists and allied health professionals). Table 1 outlines these treatments. Understanding the treatment history allows PCPs to anticipate complications and individualise care. These services are generally provided in dedicated regional and/or specialist breast units in the public sector.^8,9^ In the private sector, modes to access these services are not uniform, but treatment for breast cancer is a prescribed minimum benefit for patients who have medical insurance coverage.^10^

**TABLE 1:** Multimodal breast cancer treatment options in South Africa.

Treatment type	Specific treatment	Indications and notes	Modalities and agents available in South Africa	Common side effects
Surgery	Lumpectomy (or breast-conserving surgery)	Early-stage tumours; breast preservation; requires post-op radiation.	-	Pain, scarring, breast asymmetry, altered sensation, seroma, haematoma, cosmetic changes.
	Mastectomy	Large and/or multifocal tumours Contraindications to radiation Patient preference.	-	Chest wall numbness, tightness, phantom breast pain, seroma, shoulder stiffness, body image concerns.
	Sentinel lymph node biopsy	Clinically node-negative patients.	Uses radiotracer and/or blue dye to identify sentinel node.	Mild arm numbness, transient swelling, seroma, rare risk of lymphoedema.
	Axillary lymph node dissection	Confirmed nodal metastases or residual disease post-neoadjuvant therapy.	-	Lower lymphoedema risk than total axillary dissection, but may still cause seroma, nerve injury or arm discomfort.
	Total axillary dissection	After neoadjuvant therapy in node-positive patients.	Requires clip placement in positive node before surgery.	Lymphoedema, shoulder dysfunction, numbness, cording (axillary web syndrome), chronic pain.
	Breast reconstruction	Optional based on patient preference; immediate or delayed.	May involve implants or autologous tissue.	Implant-related complications (capsular contracture, rupture), flap necrosis, infection, asymmetry, delayed healing, altered sensation.
	Bilateral salpingo-oophorectomy	BRCA1/BRCA2 (BReast CAncer gene) mutation carriers or strong family history of breast or ovarian cancer after genetic counselling and risk assessment. Premenopausal, hormone receptor positive with contraindications to medical suppression, patient preference.	-	Surgical menopause (hot flushes, vaginal dryness, mood changes), bone loss, cardiovascular risk, fatigue, sexual dysfunction. -
Radiation therapy		Post-lumpectomy or high-risk post-mastectomy; axillary lymph node dissection alternative.	External beam radiation therapy; 3-dimensional conformal radiation therapy, intensity-modulated radiation therapy or hypofractionated regimens.	Skin changes (erythema, fibrosis), fatigue, breast and/or chest wall tightness, brachial plexopathy, lymphoedema, cardiac or lung toxicity (rare).
Systemic therapy	Chemotherapy	High-risk early-stage or metastatic disease; neoadjuvant or adjuvant.	Doxorubicin (adriamycin), cyclophosphamide, paclitaxel, docetaxel, capecitabine, carboplatin, 5-Fluorouracil.	Fatigue, nausea, alopecia, neuropathy, cognitive changes (‘chemo brain’), early menopause, infertility, myelosuppression, weight changes.
	Hormone (endocrine) therapy	Hormone receptor positive tumours; adjuvant or neoadjuvant. In frail or elderly hormone receptor positive patients unfit for surgery, this could be the primary treatment option.	Tamoxifen, letrozole, anastrozole, exemestane, fulvestrant.† Gonadotropin releasing hormone agonist (e.g. goserelin)†	Hot flushes, arthralgia, vaginal dryness, mood swings, fatigue, bone loss (with aromatase inhibitors), sexual dysfunction, insomnia.
	Targeted therapy	Human epidermal growth factor receptor 2 positive, BRCA-mutated or PIK3CA (phosphatidylinositol-4,5-bisphosphate 3-kinase catalytic subunit alpha) mutated tumours.	Trastuzumab (±emtansine†), pertuzumab,† alpelisib,† olaparib,† abemaciclib,† palbociclib,† ribociclib,† everolimus†	Cardiotoxicity (trastuzumab), fatigue, diarrhoea, rash, liver enzyme elevation, infusion reactions.
	Immunotherapy	Triple-negative breast cancer with PD-L1 (programmed death-ligand 1) expression.	Pembrolizumab,† atezolizumab†	Immune-related adverse events (e.g. thyroiditis, colitis, pneumonitis), fatigue, rash, joint pain, rare endocrinopathies (e.g. adrenal insufficiency).

*Source*: Clinical guidelines for breast cancer control and management. National Department of Health; 2018.^8^; What do PMBs cover for breast cancer? [homepage on the Internet]. Council for Medical Schemes; 2024 [cited 2025 Sept 14]. Available from: https://www.medicalschemes.co.za/what-do-pmbs-cover-for-breast-cancer/^11^; National Comprehensive Cancer Network (NCCN). Clinical practice guidelines in oncology, breast cancer [homepage on the Internet]. 2025 [cited 2025 Sept 22]. Available from: https://www.nccn.org/professionals/physician_gls/pdf/breast.pdf^12^

† , Currently not listed on the National Essential Medicine List for these indications, but may be available in the private sector.

## Follow-up and when to refer

South African guidelines^8^ recommend 3–6 monthly follow up for BCS 1–2 years post-treatment, 6–12 monthly follow up for survivors 3–5 years post-treatment and annual follow up for those > 5 years post-treatment. Level III evidence supports regular clinical breast examination and monthly breast self-examination in asymptomatic BCS.^13^

Annual mammography with or without ultrasound is recommended for patients with early and locally advanced breast cancer following treatment, especially after breast-conserving treatment.^8^ Considering the resource constraints in SA, routine imaging of the contralateral breast following mastectomy may not be feasible in the public sector because of the need to conserve imaging resources. Ongoing mammography surveillance beyond 5 years is recommended,^8^ especially for higher-risk individuals, including those diagnosed at a younger age or with a first-degree relative affected by breast cancer. Risk stratification and judicious resource allocation remain essential when tailoring post-treatment follow-up in diverse clinical settings. Other imaging, for example, computed tomography (CT), positron emission tomography (PET) and tumour markers are *not* routinely required in *asymptomatic* patients.

Echocardiography is advised for those treated with anthracyclines or trastuzumab, typically every 3 months during treatment and every 6–12 months after treatment if there is any decline in cardiac function. Bone mineral density measurement with Dual-Energy X-ray Absorptiometry (DEXA) testing is recommended every 2–3 years for BCS on AIs.

Primary care providers should vigilantly screen and examine for:

- New masses (breast, chest wall, axilla).
- Persistent bone pain or neurological symptoms.
- Weight loss or fatigue without another cause.
- Shortness of breath or a new cough.
- Abnormal blood tests (if ordered based on symptoms).

Urgent referral or imaging is warranted should any of these arise.

Other relevant cancer screening (e.g. cervical and colon cancer) should be conducted as for the general public.

## Endocrine therapy

Approximately 60% – 70% of BCS have HR+ cancer.^14,15^ Endocrine therapy reduces recurrence and mortality in HR+ breast cancer.^8^ Endocrine therapy duration depends on risk stratification and menopausal status. Standard duration of therapy is 5 years, although extended treatment for 7–10 years is recommended for some BCS (see further down). Patients with metastatic disease need to continue endocrine therapy lifelong or until there is disease progression as determined by the treating oncologist. A switch to a drug in a different class is indicated in these circumstances.^8^

Tamoxifen, a selective oestrogen receptor modulator, is approved for use in HR+ breast cancer in SA. In breast cells, it acts as an oestrogen antagonist, preventing oestrogen from binding to its receptor. This blockade inhibits tumour growth, as oestrogen normally promotes proliferation in hormone-sensitive breast cancer cells.^16^ Patients on tamoxifen should undergo an annual gynaecological examination to rule out endometrial and other gynaecological pathology.^8^

In postmenopausal women, the ovaries no longer produce significant oestrogen. Instead, oestrogen is synthesised via aromatase-mediated conversion of androgens. Aromatase inhibitors inhibit this enzyme, leading to a dramatic reduction in circulating oestrogen levels. This oestrogen deprivation starves HR+ breast cancer cells, slowing or halting their growth.^17^ The non-steroidal (reversible inhibitor) AIs, anastrozole or letrozole, are indicated for adjuvant breast cancer treatment in women with confirmed intolerance to tamoxifen, that is thrombo-embolic disease or endometrial hyperplasia (proven on ultrasound).^8^ Exemestane, a steroidal (irreversible inhibitor) AI, is often used after disease progression on non-steroidal AIs or if non-steroidal AIs are not tolerated.^8^ Aromatase inhibitors are generally considered as first-line endocrine therapy for post-menopausal women with HR+ breast cancer in the private sector in SA, reflecting international guidance.^11^

Extended endocrine therapy is typically considered for patients with a higher risk of late recurrence as determined by the specialist treating team. A recent meta-analysis^18^ suggests that extending AIs to 7 or 8 years (rather than 10 years) may offer the best balance of benefit and tolerability in postmenopausal survivors with HR+ early-stage breast cancer. However, the decision should also weigh side effects (e.g. osteoporosis, arthralgia, fatigue) and patient preference alongside oncology input.

Paracetamol, non-steroidal anti-inflammatories (NSAIDs) and exercise may be effective treatments for vasomotor and musculoskeletal side effects of AIs.^19^ Venlafaxine and clonidine are recommended for hot flushes.^8^

## Trastuzumab (Herceptin)

Trastuzumab binds to the extracellular domain of the human epidermal growth factor receptor 2 (HER2) on breast cancer cells, preventing dimerisation and downstream signalling through multiple pathways. It also activates immune-mediated cell destruction via antibody-dependent cellular cytotoxicity, reducing tumour growth and HER2 surface expression.^20^ Around 25% of early BCS have overexpression of HER2.^21^ In December 2019, the South African National Essential Medicines List Committee (NEMLC) amended the duration of trastuzumab therapy from 12 months to 6 months in the adjuvant management of early HER2-positive breast cancer.^22^ This change reflects findings from evidence that a 6-month treatment regimen is non-inferior compared to a 12-month regimen, and halves cardiovascular risks of treatment.^23^

## Bone health

Breast cancer survivors at increased risk of osteopenia and osteoporosis include those who are on AIs, undergoing ovarian suppression, have a low body mass index (BMI), smoke, or use corticosteroids.^24^ Calcium and Vitamin-D supplementation are indicated for these survivors. Bisphosphonates or denosumab and falls risk prevention strategies are indicated for those with confirmed osteopenia or osteoporosis.^8,24^

## Cardiovascular health

Breast cancer survivors at increased risk of cardiovascular complications include those with pre-existing risk factors such as smoking, high alcohol intake, obesity, and a sedentary lifestyle, as well as those of advanced age or with a family history of heart disease. Additional risk factors include hypertension, diabetes mellitus, hypercholesterolaemia, and existing cardiac conditions (e.g., heart failure, asymptomatic left ventricular systolic dysfunction, cardiomyopathy, or coronary artery disease). Survivors treated with potentially cardiotoxic therapies such as anthracyclines (e.g., doxorubicin), trastuzumab, or chest wall radiotherapy, are also at elevated risk.^8,24^ Breast cancer survivors with pre-existing or underlying cardiovascular conditions should be treated in line with existing guidelines.

## Lymphoedema

Lymphoedema occurs in up to 30% of women after axillary surgery or radiotherapy,^25^ with other risk factors including obesity, upper limb infection or trauma. It presents with upper limb swelling, heaviness or tightness, and may impair function and quality of life. Lymphoedema may develop at any stage during or after breast cancer treatment. Educating BCS on limb care such as skin hygiene, avoiding trauma, gentle exercise, infection prevention and weight management are crucial.^26^ Primary care providers should screen for early symptoms and refer to appropriately trained lymphoedema treatment providers promptly to avoid lymphoedema progression.^8^ The Lymphoedema Association of South Africa (LAOSA)^27^ maintains a national practice register of qualified lymphoedema therapists.

## Psychosocial support and health promotion

Long-term effects of cancer and its treatment can be emotionally and socially complex. Common issues include anxiety about recurrence, depression or post-treatment adjustment, cognitive difficulties and employment and financial stress.^24^ Screening for and treating depression, offering counselling or referring to social workers or mental health support, and encouraging peer support groups and survivorship education are all in the purview of PCPs. Promoting treatment adherence, exercise, smoking cessation, moderate alcohol intake and supporting dietary advice focused on whole foods and weight control, including referral to dietitians, are indicated for the holistic management of BCS.^8^ Rehabilitation is a critical component of survivorship care, aiming to restore physical function, reduce symptom burden and improve quality of life. Early integration of rehabilitation services including physical therapy, occupational therapy and exercise programmes tailored to individual needs are important to address persistent issues such as fatigue, pain and mobility limitations.^28^

## Sexual and reproductive health

Disruption of body image, sexual dysfunction and intimacy concerns are often neglected aspects of cancer care survivorship. Addressing sexual health proactively is a crucial aspect of providing breast cancer survivorship care. Assessing for reversible contributing factors and treating these when appropriate are indicated. Non-hormonal, water-based lubricants can be prescribed for vaginal dryness.^19^ Topical oestrogen can be considered after consulting the treating oncologist.^29^ Referral to sexual health support services should be considered if available. Pregnancy after treatment is often safe for younger women, and fertility preservation should be discussed before treatment begins.^24^ Survivorship care must include timely, individualised counselling on fertility options, contraception and family planning.^8^

## Coordinated survivorship care

Survivorship care plans summarising the diagnosis, treatment, and follow-up schedule may facilitate shared care between the oncology team at the tertiary level and PCPs.^30^ These may enhance continuity of care, empower patients and ensure proactive surveillance. A simple checklist for each visit may include symptom review, endocrine therapy adherence and side effect assessment, lymphoedema screening, mental health and sexual wellness review, lifestyle interventions and health promotion.

## Case vignette continued

After a comprehensive clinical consultation with Ms. R, you establish that her hypertension is well-controlled and she has no symptoms or signs of cardiovascular compromise, breast cancer recurrence, anxiety or depression. Her only complaint is joint pains in the knees and wrists in the mornings which improves during the day. She reports good adherence to letrozole. She has no signs or symptoms of lymphoedema. You establish that she is a non-smoker who attends a community exercise class 4 or 5 times per week, with an acceptable BMI. She does not currently have a sexual partner and reports no genito-urinary discomfort. You liaise with her oncologist at the tertiary centre who agrees that Ms. R is eligible for primary care follow up. The oncologist advises that Ms. R could discontinue the letrozole, having received 8 years of adjuvant endocrine treatment and considering her acceptable clinical response. Ms. R agrees to this. The oncologist advises that Ms. R continue calcium and vitamin D supplementation and undergoes a repeat mammogram and DEXA in 1 year (after reviewing reassuring recent examinations). Ms. R, reassured and fully informed of her stable clinical status, treatment history and ongoing health needs, agrees to the proposed plan through shared decision making. She is willing to undergo her repeat special investigations at the tertiary centre and understands the need for possible referral back to the tertiary centre should breast cancer recur.

## Conclusion

Breast cancer survivorship care is a growing component of the PCP’s workload. Equipped with clear guidance and practical tools, PCPs can offer high-value follow-up that not only detects breast cancer recurrence but also improves overall patient well-being. With the shift towards integrated chronic disease management in SA, breast cancer survivorship deserves structured support across the continuum of care.
